# Intratumoral bacteria in cancer: from detection to biological relevance

**DOI:** 10.3389/fonc.2026.1904204

**Published:** 2026-08-06

**Authors:** Mingjie Ding, Hao Li, Wenzhi Guo

**Affiliations:** 1 Department of Hepatobiliary and Pancreatic Surgery, The First Affiliated Hospital of Zhengzhou University, Zhengzhou, China; 2 Henan Key Laboratory of Digestive Organ Transplantation, Zhengzhou, China; 3 Henan Engineering & Research Center for Diagnosis and Treatment of Hepatobiliary and Pancreatic Surgical Diseases, Zhengzhou, China; 4 Henan Provincial Engineering Research Center for Clinical Application and Translation in Organ Transplantation, Zhengzhou, China; 5 Henan Key Laboratory for Hepatopathy and Transplantation Medicine, Zhengzhou, China

**Keywords:** intratumoral bacteria, spatial organization, tumor immunity, tumor microbiome, tumor microenvironment

## 1 Introduction

The tumor microenvironment is usually framed in cellular terms, including malignant cells, stromal elements, vascular compartments and infiltrating immune populations. Over the past decade, bacterial signals within tumor tissue have moved from an unexpected observation to a recurrent finding across cancer types. This shift has been driven by studies that go beyond bulk detection to describe intracellular localization, spatial restriction and associations with host-cell states (1). In parallel, cancer biology has increasingly recognized polymorphic microbes and the microbiome as part of the context in which tumors evolve and respond to therapy (2).

**Figure 1:**
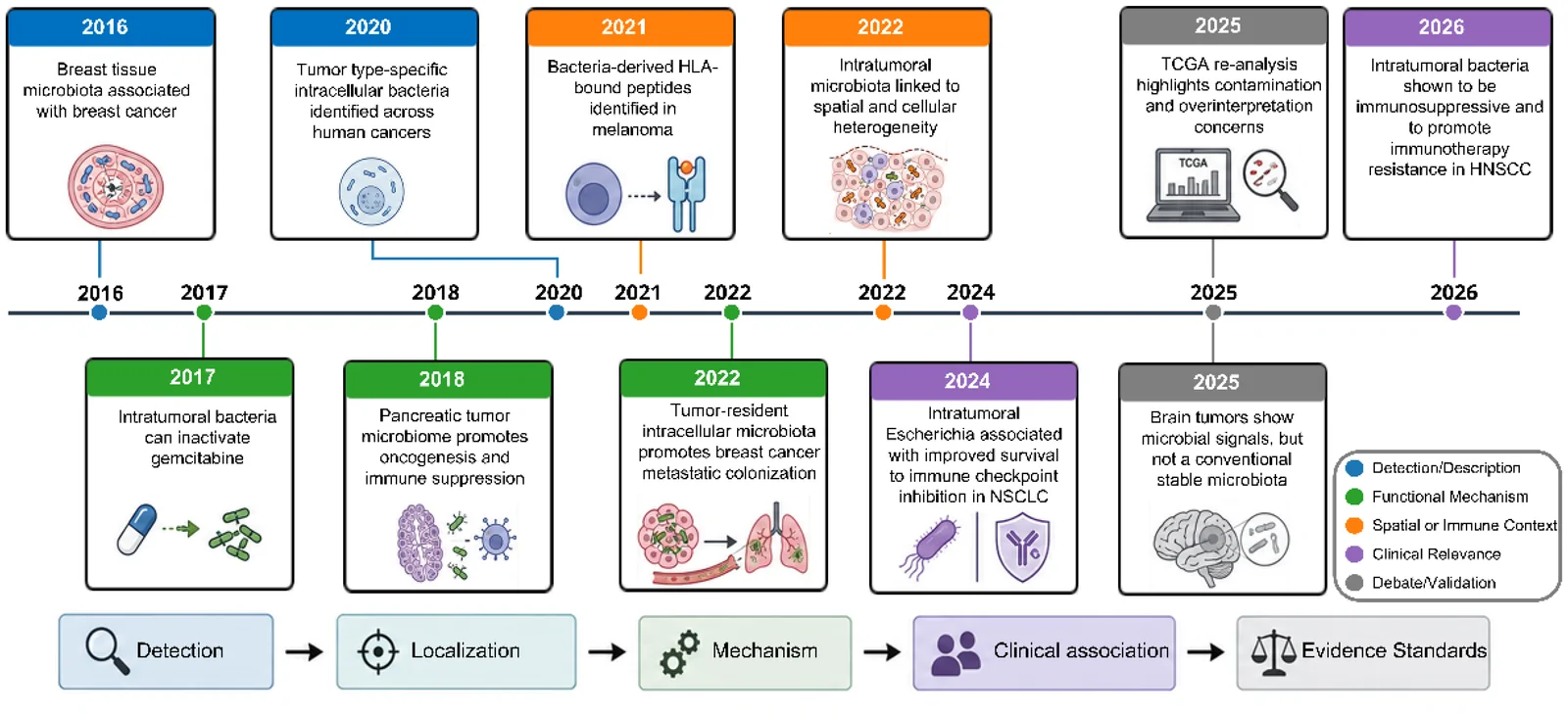
Milestones in intratumoral bacteria research. Timeline of key advances in the field, from early detection of intratumoral bacterial signals to spatial localization, functional implication, clinical association, and recent emphasis on validation and interpretive caution. The lower sequence summarizes the evidentiary progression from detection and localization to mechanistic testing, clinical association and increasingly stringent evidence standards.

The central question is therefore no longer whether bacterial material can be detected in tumors. It is when such findings should be considered biologically meaningful. This distinction matters because recent studies have linked intratumoral bacteria, or bacteria-derived components, to tissue compartmentalization, immune contexture, metastatic behavior and therapeutic response (3, 4). Yet the field remains constrained by the challenges of low-biomass microbiology, including contamination, platform bias and the gap between signal detection and causal inference. Progress has followed a clear arc: descriptive detection, spatial localization, functional implication, clinical association and, more recently, stricter validation standards and interpretive caution (**Figure 1**).

## 2 Early observations challenged tumor sterility

Early studies did not establish a unified concept of tumor microbiota, but they did challenge the long-standing assumption that internal tumors are necessarily sterile. Initial work in pancreatic cancer suggested that intratumoral bacteria could influence chemotherapeutic efficacy, and broader profiling later showed that distinct human tumor types harbor detectable and often intracellular bacterial components (5, 6). These reports were largely descriptive, showing that bacterial material can be detected within tumors. Their broader implication is that the traditional assumption of absolute tumor sterility requires re-evaluation, while debate continues regarding the prevalence, viability and stability of intratumoral bacterial communities.

These observations were not limited to one disease model or one analytic platform. Instead, they suggested that tumor-associated bacterial signals can recur across cancers, although their abundance and composition vary markedly. However, a fundamental limitation is that detection alone cannot ascertain biological relevance, particularly in scenarios characterized by low microbial biomass and indistinguishable technical errors. Confidence in this threshold also depends on adequate sequencing depth, explicit batch modeling, laboratory and reagent-specific contaminant tracking, transparent taxonomic classification, and sensitivity analyses across bioinformatic filtering strategies. Reproducible presence should therefore require negative controls, independent replication across sample sets or laboratories, batch-aware analysis, concordant microscopy or *in situ* validation, and careful distinction between bacterial DNA, RNA, antigens and viable organisms (3, 4, 7).

## 3 Spatial studies placed bacterial signals in tissue and cellular context

Subsequent work made the field more biologically compelling by placing bacterial signals in histological and cellular context. Rather than treating tumors as homogenized specimens, newer studies asked where these signals are located, which host-cell populations contain them and whether they show recognizable spatial organization. This was an important conceptual advance because it addresses the second threshold: ecological organization. Operationally, this threshold is supported when spatial transcriptomics, spatial proteomics, multiplex imaging or *in situ* hybridization reproducibly identifies bacterial signals within defined cell types or microniches rather than in diffuse or batch-specific patterns. Once bacterial signals could be mapped to tissue compartments and host-cell states, the discussion shifted from simple detection to possible tissue-level relevance (8–10), such as intracellular persistence within immune and malignant cells or the formation of extracellular polymicrobial biofilms.

Spatially resolved analyses have illustrated this shift particularly clearly. Intratumoral microbiota were not randomly distributed, but occupied discrete microniches associated with specific immune and epithelial programs (8). In breast cancer, tumor-resident intracellular bacteria were linked to metastatic colonization (9). In pancreatic cancer, tumor microbiome patterns were associated with host cellular programs and immunity (10). Together, these studies did not settle the field, but they strengthened the argument that some bacterial signals occupy nonrandom tumor microniches and are embedded in host-cell organization rather than nonspecific background.

## 4 Links to immunity, invasion and therapeutic response

More recent studies suggest that intratumoral bacteria may be connected to clinically relevant tumor behavior. Importantly, the evidentiary level differs by study design: drug metabolism, immune suppression and metastatic colonization have been tested in experimental models, whereas many links between microbial features, immune states and clinical outcomes remain observational. Functional work has shown that bacteria can inactivate gemcitabine in pancreatic cancer models and that tumor-associated microbes can contribute to immune suppression during pancreatic tumorigenesis (4, 11). Additional studies have implicated intratumoral bacteria in metastatic fitness, local immune remodeling and invasion-related phenotypes (9, 12, 13). These findings begin to address the third threshold by moving the field beyond morphology toward biological consequence (14, 15). In hepatocellular carcinoma (HCC), for instance, intratumoral microbial heterogeneity has been associated with variation in the immune microenvironment and clinical outcomes (16). The unique gut-liver axis provides a direct anatomical conduit where microbe-derived components and associated metabolites can shape hepatic local immunity, further underscoring the tissue-specific nature of these interactions.

Human observational data have added further complexity. Bacteria-derived antigens and microbial peptides can be visible to antitumor immunity in glioblastoma and melanoma (12, 17). Across lung cancer and head and neck squamous cell carcinoma, intratumoral bacterial features have been associated with divergent immunotherapy outcomes (6, 13, 18). Mechanistic commentary has therefore focused on how intratumoral microbes might either potentiate or impair anticancer immunity depending on context (19, 20). Accordingly, human associations with immune state or immunotherapy outcome should not be interpreted as causal unless supported by microbial perturbation, rescue or longitudinal clinical validation. They suggest that the biological significance of intratumoral bacteria varies across tumor types, tissue niches and treatment settings (16, 19–21).

These observations have not produced consensus. Repeated detection across cancers, increasing spatial resolution, host-cell localization and selected functional studies make it difficult to dismiss the entire field as artifact. At the same time, low microbial biomass, reagent contamination, laboratory-specific contaminants, batch effects, insufficient sequencing depth, taxonomic misclassification, bioinformatic filtering choices, sequencing noise, annotation bias and the inferential leap from detection to causality remain substantial limitations (7, 22). A bacterial readout, a 16S ribosomal RNA gene signal or even tissue-level colocalization does not necessarily establish a viable, persistent or functionally consequential bacterial population.

Recent studies of brain tumors capture this tension. A stringent multimodal analysis detected bacterial signals and intracellular microbial elements in primary and metastatic brain tumors, with associations to immune and metabolic features. Yet the same work argued against a simple interpretation in which brain tumors harbor a conventional, stable bacterial community in the usual microbiological sense (23). Similarly, a comprehensive re-analysis of microbial content in The Cancer Genome Atlas (TCGA) whole-genome sequencing data concluded that many previously claimed pan-cancer microbial findings were probably overinterpreted or technically confounded (22). The central disagreement is therefore not whether bacterial material can ever be found in tumors. It is whether the available evidence justifies biological interpretation.

## 5 Biological meaning, not detection alone

**Figure 2:**
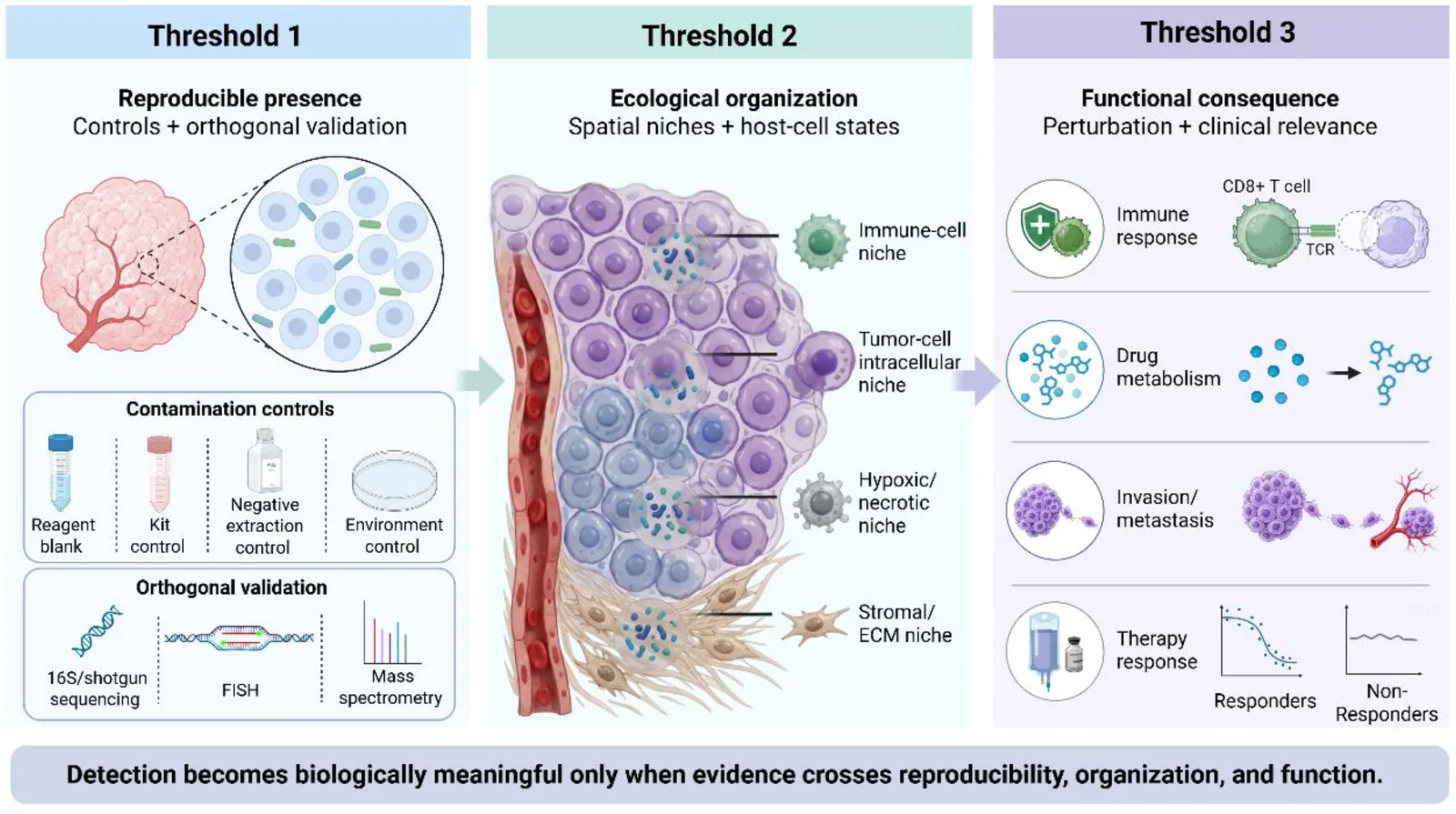
Conceptual three-threshold framework for interpreting intratumoral bacterial signals. The framework distinguishes reproducible presence supported by contamination controls, independent replication and orthogonal validation, ecological organization demonstrated by nonrandom localization within defined tissue or host-cell niches and functional consequence established by perturbation, rescue or longitudinal clinical evidence, emphasizing that detection should not be equated with causality or clinical actionability.

The framework is intended as a practical filter rather than a rigid checklist. Threshold 1 asks whether bacterial material can be reproducibly demonstrated in tumor tissue using rigorous controls and orthogonal validation. Evidence meeting this threshold would ideally include extraction and reagent blanks, independent replication and concordant microscopy, *in situ* detection or culture-independent viability assessment. Threshold 2 asks whether these signals are nonrandomly localized within defined compartments or host-cell states. Examples include reproducible localization by spatial transcriptomics, spatial proteomics, multiplex imaging or *in situ* hybridization within defined cell types or microniches. Threshold 3 asks whether bacterial presence influences tumor behavior, immune contexture, drug metabolism, invasion or treatment response; the strongest support comes from microbial depletion or enrichment, genetic or pharmacologic perturbation with rescue, or longitudinal clinical validation (**Figure 2**).

The presence and function of intratumoral bacteria should not be conflated. Some studies already move beyond detection, particularly when bacterial burden, localization or perturbation aligns with changes in immune or therapeutic phenotypes (24). In many settings, however, the data remain primarily associative and should be described at the threshold they actually reach. The field still lacks consistent evidence that bacterial signals represent viable, stable and functionally active intratumoral communities (7, 22, 23). A more rigorous vocabulary is therefore needed. Reviews and perspectives increasingly distinguish bacterial detection, spatial organization, mechanistic implication and clinical association (25–28). The present framework is intentionally restricted to bacteria and bacteria-derived signals; fungi, viruses, bacteriophages and other microbial components may require organism-specific validation criteria, even if the general progression from presence to organization to function remains adaptable.

## 6 A pragmatic reading of the evidence

A pragmatic view neither dismisses intratumoral bacteria wholesale nor elevates them prematurely into a universal feature of tumor ecosystems. The evidence is already strong enough to reject the idea that all intratumoral bacterial signals are trivial contamination (16, 21, 24). It is not yet strong enough to support the sweeping claim that bacteria are stable and biologically decisive constituents across cancers in general (14, 15, 26). The most defensible position is that intratumoral bacteria are context-dependent biological modifiers. In some tumors and clinical settings, they may alter local immune states, treatment response or metastatic behavior. In others, the evidence remains mainly descriptive.

This perspective is especially important in the era of single-cell and spatial oncology. Integrated spatial transcriptomics, spatial proteomics and high-resolution imaging can connect bacterial localization with host transcriptional and protein states, provided that contamination-aware preprocessing and orthogonal confirmation are incorporated. These approaches are valuable not only because they improve detection, but because they place bacterial signals within tissue architecture, cellular neighborhoods and local immune programs (8, 26, 29). Intratumoral bacteria may therefore be best understood as stratified tissue-level variables whose relevance depends on cancer type, microanatomic niche and therapeutic setting.

For investigators, the framework suggests a simple reporting practice: state which threshold has been met, which controls support that threshold and which causal claims remain untested. The field may already have moved beyond asking whether intratumoral bacterial signals exist. The more useful question is when those signals become biologically interpretable and clinically relevant. Intratumoral bacteria should enter mainstream models of the tumor ecosystem only when evidence crosses three thresholds: reproducible presence, ecological organization and functional consequence. Until then, the most productive stance is careful interpretation grounded in tissue context, biological plausibility and disease specificity.

## 7 Conclusions

Intratumoral bacteria have moved cancer microbiome research beyond the question of simple detection. The more important issue is whether bacterial signals are reproducible, spatially organized and functionally consequential within a defined tumor context. Current evidence supports a cautious but productive interpretation: intratumoral bacteria are not universal drivers of cancer, but context-dependent modifiers whose relevance should be judged against reproducible presence, ecological organization and functional consequence. Future progress will depend on spatial multiomics, higher-resolution imaging, single-cell microbial transcriptomics, culture-independent viability assays and standardized contamination-control pipelines that can test these thresholds reproducibly across laboratories. Their relevance is likely shaped by cancer type, tissue niche, immune state and therapeutic pressure.
